# Classification of bladder cancer cell lines according to regulon activity

**DOI:** 10.3906/biy-2107-72

**Published:** 2021-12-14

**Authors:** Aleyna ERAY, Serap ERKEK-ÖZHAN

**Affiliations:** 1 İzmir Biomedicine and Genome Center, İzmir Turkey; 2 Dokuz Eylül University İzmir International Biomedicine and Genome Institute, İzmir Turkey

**Keywords:** Bladder cancer, classification, regulon, gene regulation, neuroendocrine

## Abstract

Bladder cancer is one of the most frequent cancers and causes more than 150.000 deaths each year. During the last decade, several studies provided important aspects about genomic characterization, consensus subgroup definition, and transcriptional regulation of bladder cancer. Still, much more research needs to be done to characterize molecular signatures of this cancer in depth. At this point, the use of bladder cancer cell lines is quite useful for the identification and test of new signatures. In this study, we classified the bladder cancer cell lines according to the activities of regulons implicated in the regulation of primary bladder tumors. Our regulon gene expression-based classification revealed three groups, neuronal-basal (NB), luminal-papillary (LP), and basal-squamous (BS). These regulon gene expression-based classifications showed a quite good concordance with the consensus subgroups assigned by the primary bladder cancer classifier. Importantly, we identified *FGFR1* regulon to be involved in the characterization of the NB group, where neuroendocrine signature genes were significantly upregulated, and further β-catenin was shown to have significantly higher nuclear localization. LP groups were mainly driven by the regulons *ERBB2*, *FOXA1*, *GATA3,* and *PPARG,* and they showed upregulation of the genes involved in epithelial differentiation and urogenital development, while the activity of *EGFR*, *FOXM1*, *STAT3,* and *HIF1A* was implicated for the regulation of BS group. Collectively, our results and classifications may serve as an important guide for the selection and use of bladder cancer cell lines for experimental strategies, which aim to manipulate regulons critical for bladder cancer development.

## 1. Introduction

Bladder cancer is a heterogeneous group of tumors, where transitional cell carcinoma constitutes the great majority of the cases. Classically, bladder cancer is diagnosed in two histopathological classes as ‘muscle invasive bladder cancer (MIBC)’ and ‘non-muscle invasive bladder cancer (NMIBC)’ with different prognostic and molecular characteristics (Jin et al., 2014). In the last decade, there have been a number of studies characterizing the genomic landscape of both MIBC and NMIBC and defining the molecular subgroups (Cancer Genome Atlas Research 2014; Hedegaard et al., 2016; Robertson et al., 2017; Tan et al., 2019). A more recent study aimed to define the consensus subgroups of MIBC using the gene expression data in combination with several studies (Kamoun et al., 2020), where the six consensus subgroups were referred to as ‘luminal papillary’, ‘luminal nonspecified’, ‘luminal unstable’, ‘stroma-rich’, ‘basal/squamous’, and ‘neuroendocrine-like’. In this study, the authors, in addition, associated these subgroups with distinct regulon activities, previously defined in (Robertson et al., 2017). These regulons implicated in bladder carcinogenesis include transcription factors and growth factor receptors, determined according to their gene regulatory activity in bladder cancer (Robertson et al., 2017). 

Bladder cancer cell lines have been extensively used for modeling the development, progression and molecular characteristics of bladder cancer. In addition to the focused characterization of cell lines, where only two/three of them are used (Piantino et al., 2010; Pinto-Leite et al., 2014), there are a few other studies, which provided details about the molecular and genomic characterization of bladder cancer cell lines collectively. In one study, a classification based on the subgroups defined by (Sjodahl et al., 2012), ‘“Urobasal A”, “Urobasal B”, “Genomically Unstable”and “SCC-like” were established for 40 bladder cancer cell lines (Earl et al., 2015). Another study performed exome sequencing for 25 bladder cancer cell lines and identified the frequently mutated genes among analyzed cell lines (Nickerson et al., 2017). A more recent study provided a comprehensive review about molecular characteristics, origin, and tumorigenic properties of more than 150 murine and human bladder cancer cell lines (Zuiverloon et al., 2018). In addition, the Cancer Cell Line Encyclopedia of the Broad Institute (CCLE database) provides a unique source for the transcriptomic and genomic data produced in a variety of cancer cell lines including bladder cancer (Barretina et al., 2012). 

Although regulon activities have been significantly associated with primary bladder cancer subgroups (Robertson et al., 2017; Kamoun et al., 2020), there has not been yet a study, which characterized the bladder cancer cell lines according to regulon activities defined for the primary bladder cancers (Robertson et al., 2017; Kamoun et al., 2020). In this study, we classified the bladder cancer cell lines into 3 groups according to their regulon activities and associated the upregulated genes in each cell line group with the targets of the regulons. Our results reveal previously unknown cooperative regulatory activities in bladder cancer cells and can serve as a guide for modeling bladder cancer according to different regulon activities. 

## 2. Methods

### 2.1. Experimental methods

#### 2.1.1. Cell culture

The two bladder cancer cell lines 5637 and RT112 were obtained from DSMZ and J82 was kindly provided by Dr. S. Senturk (Izmir Biomedicine and Genome Center, Izmir). 5637 and RT112 were cultured in RPMI 1640 (Gibco BRL), J82 was cultured in DMEM (Dulbecco’s Modified Eagle Medium). All media were supplemented with %10 FBS and %1 Penicillin-Streptomycin. Cells were cultured at 37 °C and 5% CO_2_. 

#### 2.1.2. Immunofluorescence 

In 24 well plates, J82 was plated 10000/well, RT112 was plated 20000/well, 5637 was plated 40000/well. Cells were incubated overnight on glass coverslips and rinsed with 1x PBS the following day. Cells were fixed with 4% formaldehyde for 15 min at RT, and 0.2% TritonX was used for permeabilization. Fixed cells were blocked with 2% Donkey serum for 45 min. Afterwards, cells were incubated with β-catenin antibody (1:100, #9562, Cell Signaling) diluted in 2% donkey serum overnight at 4°C. Next day, cells were rinsed 2 times with 1x PBS. Goat Anti-Rabbit Alexa Fluor 594 was used as a secondary antibody. DAPI was used for nucleus staining. Coverslips were mounted onto slides for imaging with Zeiss LSM880. Images were acquired as Z-stack using ZEN 2 software. Images with maximum intensity were used for further analysis. Quantification of the images were done with ImageJ program. Splitted DAPI channel images were used to determine region of interests for nuclear β-catenin signal intensities. A total of 17 cells per cell line were used for quantification. Integrated Density Values (IDV) were used for statistical analysis. 

### 2.2. Data acquisition

CCLE RNAseq gene expression data for bladder cancer cell lines (RPKM) were downloaded from Cancer Cell Line Encyclopedia (CCLE) database (Barretina et al., 2012) and were accessed at cbioportal (Cerami et al., 2012; Gao et al., 2013). Regulon definitions were based on (Robertson et al., 2017; Kamoun et al., 2020). Mutation data for bladder cancer cell lines were obtained using cbioportal (Cerami et al., 2012; Gao et al., 2013). Neuroendocrine differentiation gene definitions are based on the information provided in Supplementary Table 3 from (Kamoun et al., 2020).

### 2.3. Data analysis

#### 2.3.1. Clustering of the cell lines according to regulon expression levels

Using the gene expression values for the regulon genes, we clustered 25 bladder cancer cell lines using kmeans option (k = 6), within pheatmap package (Kolde 2019). Only the regulons that have min 1 rpkm (log2 scale) expression value in at least one cell type analyzed were included in clustering. This resulted in 19 number of regulons which contributed to the clustering analysis. 

#### 2.3.2. Consensus classification of bladder cancer cell lines

**Figure 1:**
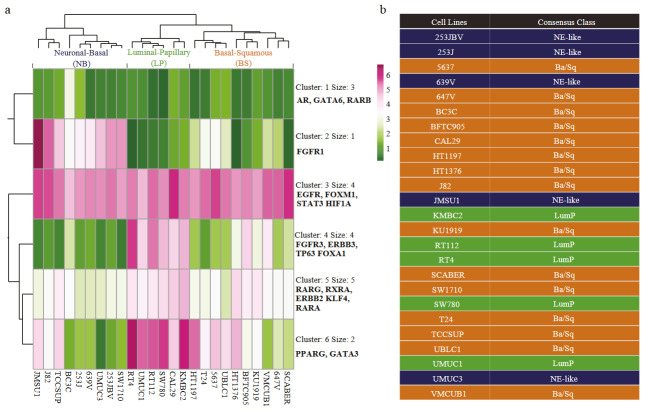
Clustering of bladder cancer cell lines according to regulon expressions (a) Heatmap visualization for the k-means clustering (k = 6) of regulon expressions in bladder cancer cell lines. Three cell line groups were represented as follows: the first group defined as Neuronal-Basal (NB), the second group defined as Luminal-Papillary (LP), the third group defined as Basal-Squamous (BS). (b) Consensus class assigned to bladder cancer cell lines. The table shows the consensus classes of the cell lines (output from the classifier for muscle invasive bladder cancer (Kamoun, et al. 2020).

In order to determine the consensus classification of bladder cancer cell lines, we utilized the “Molecular Classification of Bladder Cancer” classifier developed by Kamoun et al., (Kamoun et al., 2020) (134.157.229.105:3838/BLCAclassify). Gene expression matrix for the cell lines in rpkm (obtained from CCLE database (Barretina et al., 2012)) was uploaded to the classifier and resulting consensus classifications are presented in Figure 1b and Supplementary Table S1.

#### 2.3.3. Differential gene expression analysis

Differential gene expression analysis, where one cell line group was compared with the other groups, was performed using cbioportal (Cerami et al., 2012; Gao et al., 2013). Basically, custom cell line groups were formed based on our classifications (Figure 1), and differentially expressed genes were identified using ‘Compare’ and ‘mRNA’ options. Upregulated genes were defined using q value threshold of 0.1 and log Ratio of 0.5. 

#### 2.3.4. Gene ontology analysis and visualization

Gene ontology analysis for the upregulated gene sets was performed using the ConsensusPathDB (CPDB) database of Max Planck Institute (Kamburov et al., 2009; Kamburov et al., 2011). Overrepresentation function of the CPDB was used, and only Level 4 GO terms (Biological Process) were included for further analysis. “GOChord” function of “GOplot” R package was used for visualization (Walter et al., 2015). In chord graphs, maximum top 20 GO terms with adjusted p-value <0.05 were shown. For the limit parameter of the “GOChord” function, a minimum number of genes belonging to a specific GO term was determined as 5 if the number of the genes in upregulated gene set was >100, otherwise the number was set as 4 genes minimum. Genes, which are linked with at least 4 different GO terms, were displayed on the plots together with their logFC value representations. 

#### 2.3.5. Association of differentially expressed genes with the target genes of regulons

**Figure 2:**
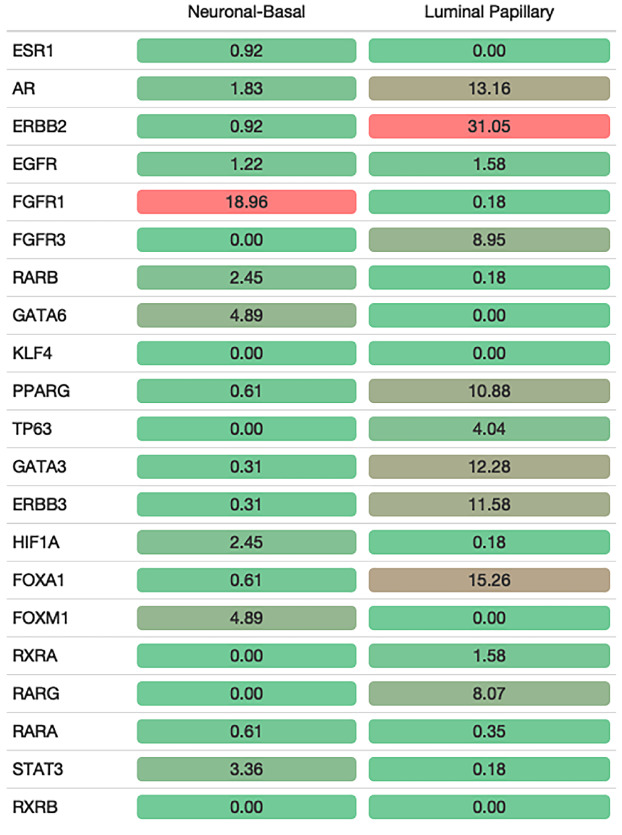
Concordance of upregulated genes in cell line groups with regulon targeting. Percentages of NB and LP upregulated genes intersecting with regulon target genes. Intersection rates are displayed from red to green (red: high, green: low).

Regulon – target gene association table was downloaded from (Robertson et al., 2017) (Table S2.25) (Robertson et al., 2017). Genes, which are positively associated with the regulons (having value=1), were referred to as the target of the respective regulons. Afterwards, upregulated genes for each cell line group were intersected with the targets of the regulons and the results were presented as percent intersection rate (Figure 2). 

### 2.4. Statistical analysis

**Figure 3:**
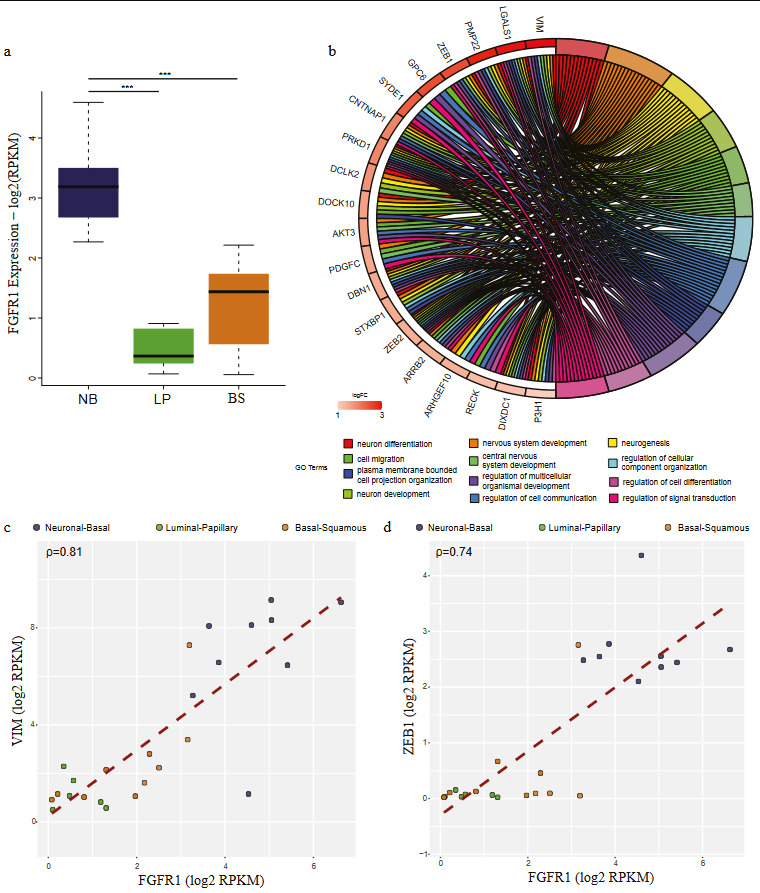
FGFR1 targets upregulated in NB group are involved in neuronal differentiation. (a) Boxplot comparing the expression of FGFR1 in three cell line groups: Neuronal-Basal (NB) (dark blue), Luminal-Papillary (LP) (green) and Basal-Squamous (BS) (orange) (ANOVA p-value=1.24e-07). Bonferroni post-hoc test was used for statistical analysis (*p < 0.05; **p < 0.01; ***p < 0.001). (b) Chord plot visualization of GO term analysis applied to the genes upregulated in NB group cell lines and intersecting with FGFR1 regulon targets. The right part of the chord plot represents the go terms, and the left part represents the genes linked with the respective terms. Genes are colored according to their logFC values. (c-d) Scatter plots comparing the expression FGFR1 with its target genes VIM (ρ = 0.81) (c) and ZEB1 (ρ = 0.74) (d). Colors represent the cell line groups.

**Figure 4:**
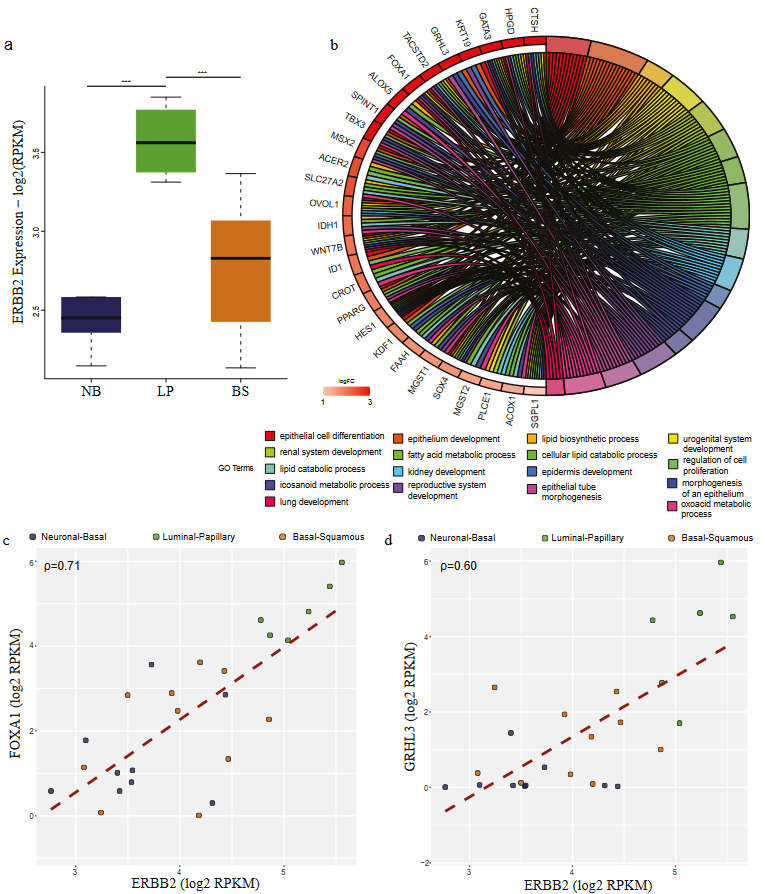
Targets of ERBB2 upregulated in LP group are implicated in epithelial morphogenesis. (a) Boxplot comparing the expression of ERBB2 in three cell line groups: Neuronal-Basal (NB) (dark blue), Luminal-Papillary (LP) (green), and Basal-Squamous (BS) (orange) (ANOVA p-value=2.36e-05). Bonferroni post-hoc test was used for statistical analysis (*p < 0.05; **p < 0.01; ***p < 0.001). (b) Chord plot visualization of GO term analysis applied to the genes upregulated in LP group cell lines and intersecting with ERBB2 regulon target genes. The right part of the chord plot represents the go terms, and the left part represents the genes associated with the terms. Coloring of the genes is done according to their expression of logFC values. (c-d) Scatter plot showing the correlation between the expression of ERBB2 and its targets FOXA1 (c) (ρ = 0.71) and GRHL3 (ρ = 0.60) (d).

Statistical analyzes were performed utilizing the R/Bioconductor packages (www.bioconductor.org). ANOVA was used to check the statistical difference among the groups for Figures 3a, 4a, 5a, and Supplementary Figure S2. Subsequently, Bonferroni post-hoc test was applied to the results of ANOVA test. Spearman correlation test was applied for Figures 3c, 3d, 4c, 4d, and 5b. Dunnett’s multiple comparisons test was used for statistical analysis of the immunostaining images (Figure 6b). 

**Figure:**
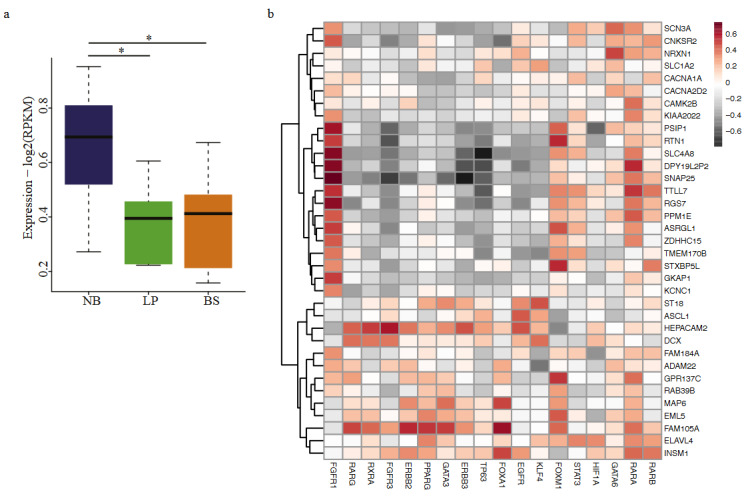
Expression profile of neuroendocrine marker genes in NB group (a) Boxplot shows the expression profile of genes associated with neuroendocrine differentiation (Kamoun, et al. 2020) in three cell line groups: Neuronal-Basal (NB) (dark blue), Luminal-Papillary (LP) (green) and Basal-Squamous (BS) (orange). (ANOVA p-value=0.0146). Bonferroni post-hoc test was used for statistical analysis (*p < 0.05; **p < 0.01; ***p < 0.001). (b) Heatmap displaying the correlation between the expression of genes involved in neuroendocrine differentiation and expression of regulons.

**Figure:**
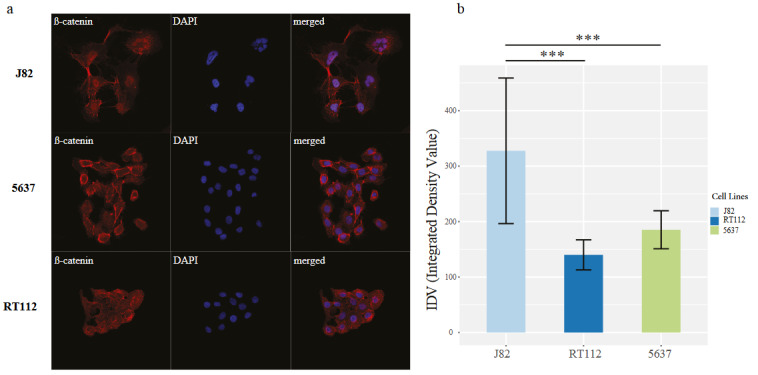
Immunostaining profile of β-catenin in cell line groups (a) Immunofluorescence images showing the staining of β-catenin cells; J82, 5638, and RT112. DAPI (blue) and β-catenin (red). (b) Barplot shows the quantification of nuclear signal in IF stainings. Dunnett’s multiple comparisons test was used for statistical analysis (*p < 0.05; **p < 0.01; ***p < 0.001).

## 3. Results

### 3.1. Grouping of bladder cancer cell lines according to regulon activity

We determined the expression of the regulon genes in 25 bladder cancer cell lines and classified these cell lines according to the expression profile of the regulon genes. Our unsupervised clustering analysis using kmeans (k = 6) clustered the bladder cell lines into 3 groups (Figure 1a). In order to find out to what extent our regulon-based classifications are legitimate, we additionally classified the cell lines using the consensus classifier algorithm provided in (Kamoun et al., 2020). This analysis identified 5 out of 9 cell lines in group 1 to be assigned to neuroendocrine-like subgroup; 6 out of 6 cell lines in group 2 were identified to belong to luminal papillary and 10 out of 10 cell lines in group 3 as basal-squamous (Figure 1b). Among the group 1 cell lines, one cell line (J82) had almost equal annotation scores (0.383 vs 0.385) for neuroendocrine-like and basal squamous classes, and, for two of the cell lines (SW1710 and TCCSUP), annotation scores were rather close as well (Supplementary Table S1). Therefore, we named the group 1-3 as ‘neuronal-basal (NB)’, ‘luminal papillary (LP)’ and ‘basal squamous (BS)’, respectively.

Although luminal and basal terms are classically used for bladder cancer cell lines (Choi et al., 2014; Zuiverloon et al., 2018), our regulon expression-based analysis here brought additional features, characteristics of each group. Our analysis revealed that the expression status of *FGFR1*, which is highly enriched in ‘stromal-rich’ subgroup in consensus classification of bladder cancer (Kamoun et al., 2020), mainly separates the NB group from the two other groups. The regulon cluster 4 driven by the expression of *FGFR3*, *ERBB3*, *TP63,* and *FOXA1* was mainly enriched for LP class; regulon cluster 6 constituted by *PPARG* and *GATA3* expression was enriched in LP class and partially in BS class. Regulon cluster 5, driven by luminal-papillary markers *RARG*, *RXRA* (Kamoun et al., 2020) and basal marker *KLF4* (Kamoun et al., 2020) was relatively enriched in LP class, with partial enrichments in NB and BS classes. Regulon cluster 3, dominated by the basal markers, *EGFR*, *FOXM1*, *STAT3* ,and *HIF1A* (Kamoun et al., 2020) were similarly enriched in all cell line groups. 

### 3.2. Differential gene expression in bladder cell line groups and association with regulon activity 

For each of the 3 groups, we determined with the clustering analysis (Figure 1a), we performed differential gene expression analysis contrasting one group with all other groups and determined the upregulated genes for each group. This analysis identified 327 and 570 upregulated genes in NB and LP classes, respectively. However, within the significance thresholds we used, we failed to detect upregulated genes for the BS class. The reason behind this can be attributed to the heterogeneous structure of this group, as it can be seen in the heatmap (Figure 1a) and in PCA analysis (Supplementary Figure S1) as well.

Having determined the upregulated genes in different cell line groups we defined, next, we tempted to relate those genes with the regulon targets. We identified the genes positively associated with the regulons using the information provided in (Robertson et al., 2017). This analysis showed that cell line groups constituted according to regulon expression profiles were in concordance with the regulon activity. For the NB group, upregulated genes had the highest intersection rate with *FGFR1* targets (18.96%), followed by *GATA6* (4.89%) and *FOXM1* (4.89%) (Figure 2). *FGFR1* was also significantly upregulated in the NB group (Figure 3a). *FGFR1* targets, which are upregulated in the NB class were mainly involved in neurogenesis, neuron differentiation, nervous system development (Figure 3b). Further, expression of the genes *VIM* and *ZEB1* implicated in epithelial to mesenchymal transition (Takeyama et al., 2010; Pluciennik et al., 2015; Larsen et al., 2016; Wu et al., 2018), highly correlated with the expression of *FGFR1*, emphasizing the role of this regulon in the transcriptomic constitution of the NB group (Figure 3c-3d).

Upregulated genes in the LP class mainly intersected with *ERBB2, FOXA1, PPARG, ERBB3, FGFR3, RARG,* and *GATA3* targets (Figure 2). We identified that almost all these regulons were significantly upregulated in the LP class (Figure 4a, Supplementary Figure S2). Target genes of the regulons upregulated in LP class were involved in epithelial cell differentiation, cell junction organization, and urogenital system development (Figure 4b, Supplementary Figure S2). Remarkably, expressions of *FOXA1* (ρ = 0.71) and *GRHL3* (ρ = 0.60) significantly correlated with the expression of *ERBB2* (Figure 4c-4d), indicating the luminal characteristics of the LP group. 

### 3.3. Cell lines belonging to NB-group expresses neuroendocrine differentiation marker genes

Our finding, which shows the enrichment of neurogenesis-related genes in the *FGFR1* targets upregulated in the NB group, prompted us to decipher this connection in more detail. As *FGFR1* is the main player characterizing this group, we checked the enrichment of *FGFR1* regulon activity in each consensus subgroup of primary bladder cancer (Kamoun et al., 2020). We discovered that although *FGFR1* has the highest enrichment score in stromal-rich consensus subgroup (Fisher’s test p-value=4.20E-41), it was also moderately enriched in neuroendocrine-like subgroup (Fisher’s test p-value= 3.18E-04) (Based on the information from Supplementary Table 3, (Kamoun et al., 2020)). To strengthen this association further, we checked the expression of genes marker of neuroendocrine differentiation (Kamoun et al., 2020) in the cell line groups we determined. This analysis also revealed that genes involved in neuroendocrine differentiation were significantly higher expressed in NB group (p-value=0.0146) (Figure 5a). Additionally, expression of *FGFR1* highly correlated with the expression of neuroendocrine markers (Figure 5b). Collectively, these results highly argue for the neuronal characteristics of the NE group and involvement of *FGFR1* in this signature. 

### 3.4. J82 cells belonging to NB group show nucleocytoplasmic staining of β-catenin

We recently showed that the WNT/β-catenin pathway is associated with the active regulatory elements characterizing neuronal bladder cancer (Eray et al., 2020). Within this frame, to check any connection of the NB group with WNT/β-catenin pathway deregulation, we scanned the cell lines we used in this study for the mutation status of β-catenin and β-catenin destruction complex components. Among the NB group cell lines, 3 of them had *APC* mutation and one had *CTNNB1* mutation. On the contrary 2 had *APC* or *CTNNB1* mutation in the two other cell line groups (Supplementary Figure S3). Based on this information, we checked the β-catenin localization in one of the NB group cell lines we had in lab J82 and the other two cell lines, 5637 (BS group) and RT112 (LP group) as controls (no mutation in *CTNNB1* or *APC*). The staining of β-catenin in 5637 and RT112 was concentrated at the cytoplasm and the membrane while in J82 it was concentrated at the nucleus of the cells. Our data showed that β-catenin showed significantly higher nuclear localization in J82 compared to the other two cell lines (Figure 6a-6b). This finding strengthens our conclusions about the involvement of WNT/β-catenin pathway in neuronal differentiation of bladder cancer cells. The information we provide for the potential involvement of FGFR1 in neuroendocrine features of bladder cancer (Figure 5), identification of significantly increased nuclear localization of β-catenin in a cell line belonging to NB group (Figure 6) collectively strengthens the neuronal/neuroendocrine characteristics of the cell lines present in NB group according to our classifications. 

## 4. Discussion

Bladder cancer cell lines serve as important models for modeling bladder tumorigenesis, invasive characteristics and treatment responses (Brown et al., 1990; Makridakis et al., 2009). So far, several studies characterized the genomic and transcriptomic properties of bladder cancer cell lines (Earl et al., 2015; Nickerson et al., 2017). In this study, we aimed to characterize the bladder cancer lines in terms of their regulon activity, defined for the primary bladder cancers in literature (Robertson et al., 2017; Lindskrog et al., 2021). Our results showed that bladder cancer cell lines have differential regulon activities, reflecting their transcriptomic signatures and their consensus classifications (Kamoun et al., 2020). 

Genes significantly upregulated in cell lines belonging to the NB group were mainly intersected the targets of *FGFR1* and were involved in neuronal differentiation. Accordingly, the expression of the genes marker of neuroendocrine differentiation (Kamoun et al., 2020) was significantly higher in the NB group compared to the two other cell line groups. In literature, *FGFR1* has been shown be expressed at higher levels in bladder cancers showing mesenchymal features (Cheng et al., 2013). Knock-down of *FGFR1* in JMSU1 and UMUC3 cell lines, belonging to NB group in our results, resulted in a significant reduction in the anchorage-independent ability of these cells (Tomlinson et al., 2009). Further *FGFR1* expression was high in most small cell carcinoma of the bladder (Yang et al., 2020), which is a rare type of bladder cancer with neuroendocrine differentiation (Ghervan et al., 2017; Wang et al., 2019). These existing literature and our findings highly support the association of *FGFR1* with NB characteristics and neuronal differentiation of bladder cancer. 

We previously showed that WNT/β-catenin pathway is deregulated in neuronal subtype of bladder cancer (Eray et al., 2020). In this study, we identified significantly higher accumulation β-catenin in nucleus in J82 cell line belonging to NB group, which has a mutation in *APC*, a component of β-catenin destruction complex (Krishnamurthy and Kurzrock 2018; Parker and Neufeld 2020). It is known that the immune gene expression signature is relatively depleted from small cell neuroendocrine carcinoma of the bladder (Yang et al., 2020), and neuroendocrine-like bladder cancer show decreased levels of immune infiltrate (Kamoun et al., 2020). It was also identified that Wnt/β-catenin signaling can decrease the T-cell infiltration in melanoma mouse models. Thus, inhibition of Wnt signaling has been suggested to prevent immunotherapy resistance (Chehrazi-Raffle et al., 2021). In addition, inhibition of *FGFR1* has been shown to enhance the immune checkpoint inhibitor response in breast cancer (Akhand et al., 2020). Based on all these information, we checked the expression of *CXCL16*, T cell chemoattractant (Akhand et al., 2020) in bladder cancer cell lines and identified a significant negative correlation with *FGFR1* expression (Supplementary Figure S4). Our data and existing literature together suggest a regulatory axis involving *FGFR1*, WNT/ β-catenin signaling, and tumor immune microenvironment in regulation of NB cell lines. Therefore, we suggest that combinatorial treatment strategies disrupting this regulatory axis can be applied on NB cell lines. 

Regulons implicated in LP group cell lines are mainly known for early bladder cancer, mostly non-muscle invasive and luminal associations. *ERBB2* has been identified to be overexpressed in high-risk non-muscle invasive bladder cancer (Hedegaard et al., 2016) and as one of the major prognostic factors for survival status of the patients (Cormio et al., 2017; Moustakas et al., 2020). *FOXA1* expression was adequate for separating non-basal subtype of bladder cancer from the basal subtype (Sikic et al., 2020). Furthermore, *GATA3*, *FOXA1,* and *PPARG* have been shown to drive the luminal fate in a collaborative manner (Warrick et al., 2016). Thus, within this frame, our regulon-based classifications confirm the luminal character of the LP class we defined. 

Our differential gene expression analysis did not identify significantly upregulated genes in the BS class, largely because of the heterogeneity of this group (Supplementary Figure S1). However, we determined *EGFR, FOXM1 and STAT3* as the main regulons, driving the basal characterization of this group (cluster 3, Figure 1a). *EGFR* has been previously shown to be enriched in basal-like bladder cancer, and some groups of muscle invasive bladder cancers have been determined to respond to *EGFR* inhibitors (Rebouissou et al., 2014). In addition, expression of *FOXM1* as a prognostic factor in the survival of muscle invasive bladder cancer patients (Rinaldetti et al., 2017), *STAT3* expression, and phosphorylation was identified to be substantially higher in basal-like bladder cancer (Gatta et al., 2019). Further, *STAT3* activated transgenic mice directly developed invasive bladder cancer without going through the intermediate noninvasive stages (Ho et al., 2012). Our results here collectively emphasize the role of *EGFR*, *FOXM1,* and *STAT3* in basal characteristics of BS cell lines. 

To conclude, our regulon-based classification of bladder cancer cell lines may serve as an important guideline for studying the different regulons implicated in bladder cancer and trial of drug candidates relevant for targeting regulons. 

## Authorship contribution statement

**Aleyna Eray: **Design of the study, computational and experimental analysis, writing of the manuscript. 

**Serap Erkek-Ozhan: **Design, supervision of the study, writing of the manuscript.
